# Plasma from pre‐eclamptic patients induces the expression of the anti‐angiogenic miR‐195‐5p in endothelial cells

**DOI:** 10.1111/jcmm.12767

**Published:** 2016-03-24

**Authors:** Valeria Cristina Sandrim, Mayara Caldeira Dias, Ana Lívia de Carvalho Bovolato, Jose E Tanus‐Santos, Elenice Deffune, Ricardo C. Cavalli

**Affiliations:** ^1^ Department of PharmacologyInstitute of Biosciences of BotucatuUniversidade Estadual Paulista (UNESP)BotucatuSPBrazil; ^2^ Blood Transfusion CenterCell Engineering LaboratoryBotucatu Medical SchoolSão Paulo State University (UNESP)BotucatuSPBrazil; ^3^ Department of PharmacologyFaculty of Medicine of Ribeirao PretoUniversity of Sao PauloRibeirao PretoSPBrazil; ^4^ Department of Gynecology and ObstetricsFaculty of Medicine of Ribeirao PretoUniversity of Sao PauloRibeirao PretoSPBrazil

**Keywords:** pre‐eclampsia, microRNA, angiogenesis, miR‐195, HUVECs, VEGF

## Abstract

We examined the effect of plasma incubation from preeclampsia pregnant on the antiangiogenic miR‐195‐5p expression. Higher miR‐195‐5p expression was found in cultures incubated with preeclampsia plasma compared to those incubated with healthy pregnant plasma. Next, as VEGF is a target of miR‐195‐5p we have quantified its expression by real‐time qPCR and ELISA. We found reduced VEGF levels in culture incubated with preeclampsia plasma. Therefore, we have concluded that the higher expression of miR‐195‐5p in endothelial cell cultures incubated with preeclampsia plasma may contribute to decreased expression of VEGFA (gene and protein) and increased antiangiogenic status in preeclampsia. Therefore, this miR may be an important target in preeclampsia.

## Introduction and objective

Down‐regulation of miR‐195‐5p has been associated with increased angiogenesis in human hepatocellular carcinoma 1. This effect is partially mediated by the binding of miR‐195‐5p in the 3′untranslated region (UTR) of vascular endothelial growth factor (*VEGFA*), leading to decreased VEGFA expression 1. VEGFA is an important molecule involved with vasodilation, proliferation, permeability, migration and survival of endothelial cells 2. Interestingly, elevated miR‐195‐5p concentrations were found in placenta from pre‐eclamptic compared to healthy pregnancy 3, and it is well known that pre‐eclampsia is characterized by an anti‐angiogenic status, including high levels of sFLT‐1 (soluble fms‐like tyrosine kinase‐1) and sEng (soluble endoglin), which contrast with low free VEGFA concentrations 2, 4, 5.

Generalized maternal endothelial dysfunction is central to the pathophysiology of pre‐eclampsia, contributing to the most relevant features of the disease: hypertension and proteinuria 6, 7, 8. Several studies have reported a relationship between maternal endothelial dysfunction and factors present in plasma/serum of pre‐eclamptic pregnant 9, 10, 11. Those circulating factors are in part released by ischaemic placental tissue into maternal circulation, and target endothelial cells causing alterations in their function 8, 11. Supporting this concept, endothelial cells incubated with plasma/serum from pre‐eclamptic pregnant undergo *in vitro* modifications 10, 12, such as increased expression of the vasoconstrictor endothelin‐1 13, 14.

The molecular mechanisms by which factors present in plasma/serum induce dysfunction of endothelial cells are not completely understood. We suggest here that epigenetic processes mediated by miR could contribute to these modifications. Therefore, our hypothesis is that endothelial cells incubated with plasma from pre‐eclamptic pregnant present higher expression of miR‐195‐5p which may decrease VEGFA expression and contribute to the anti‐angiogenic status of the disease. Therefore, this study aimed at comparing the expression of miR‐195‐5p and *VEGFA* in human umbilical vascular endothelial cells (HUVECs) incubated with plasma from pre‐eclamptic and healthy pregnant.

## Study design

This study was approved by Institutional Review Board of the *Hospital Santa Casa*, Belo Horizonte, Minas Gerais State, Brazil (Reference number 035/2009) and complies with the principles of the Declaration of Helsinki. Plasma from pre‐eclamptic (*n* = 7) and healthy pregnant (*n* = 10) were incubated (20% v/v) with HUVECs (ATCC‐CRL 2873) for 24 hrs at 37°C, 5% CO_2_. The groups were matched, respectively to healthy pregnancy and pre‐eclampsia, to gestational age at sampling (35 ± 3 *versus* 34 ± 4 weeks), maternal age (24 ± 6 *versus* 28 ± 6 years) and body mass index (32 ± 5 *versus* 33 ± 7 kg/m^2^). Systolic and diastolic blood pressure were significantly elevated in pre‐eclampsia compared to healthy pregnant (133 ± 12 *versus* 113 ± 9 and 71 ± 7 *versus* 86 ± 10 mmHg, respectively, *P* < 0.05).

Total RNA were extracted from HUVECs using miRNeasy Mini Kit according to the protocol of the manufacturer. Quantification and purity of isolated RNA was performed using the NanoDrop Spectrophotometer (Thermo Scientific, Waltham, MA, USA) and it was consistently found to be pure. miR‐195‐5p and the reference U6 were quantified by miScript SYBR Green qRT‐PCR assays using specifics primers from Qiagen^®^, Leusden, Netherlands. *VEGFAA* expression was performed using the KiCqStart Universal SYBR Green qPCR (Sigma‐Aldrich, Poole, UK) by qRT‐PCR. The *HPRT1* gene was chosen as endogenous control as it was the most stable housekeeping gene in the samples based on the housekeeping genes panel from the RT^2^ RNA QC PCR Array (Qiagen^®^). Relative quantification was calculated using the 2^−ΔCq^ method and normalization was performed to U6 or *HPRT1*. Each sample was performed in duplicate in all PCR reactions. The threshold cycle (C_T_) refers to the fractional cycle number at which the fluorescence passes the fixed threshold. Also, we have measured VEGFA levels in supernatant of cell culture using ELISA kit (RAB0507; Sigma‐Aldrich^®^).

Student′s *t*‐test was used to compare clinical data and *VEGFA* gene expression (2^−ΔCq^) between pre‐eclampsia and healthy pregnancy. Mann–Whitney test was used to compare VEGFA levels in supernatant and miR‐195‐5p expression (2^−ΔCq^) between groups. These statistical analyses were performed using GraphPad Prism 5.0 (GraphPad Software, San Diego, CA, USA). Gene and miRNA expression data were analysed by GeneGlobe Data Analysis Center (Qiagen^®^) online platform. For all tests, *P*‐value <0.05 (two‐tailed) was considered significant.

## Results and discussion

**Figure 1:**
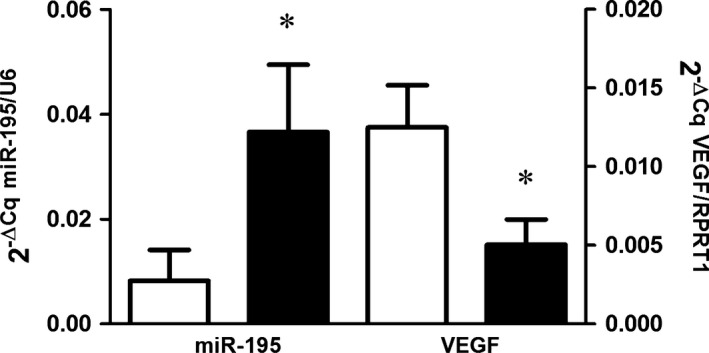
Expression levels of miR‐195/U6 and *VEGFAA/HPRT1*(mRNA) in HUVECs incubated with plasma from healthy pregnancy (HP) and pre‐eclampsia (PE). *P ≤ 0.05 compared to healthy pregnancy.

We found increased miR‐195 expression in HUVECs incubated with plasma from pre‐eclamptic pregnant as compared those incubated with plasma from healthy pregnant (6.7‐fold increase, and 2^−ΔCq^ of 0.042 ± 0.031 *versus* 0.008 ± 0.003, respectively, *P* < 0.01; Fig. 1) Additionally, HUVEC incubated with plasma from pre‐eclamptic pregnant showed reduced *VEGFA* expression as compared to those incubated with plasma form healthy pregnant (−2.43‐fold decrease, and 2^−ΔCq^ of 0.005 ± 0.004 *versus* 0.012 ± 0.008 respectively, in pre‐eclamptic *versus* healthy; *P* = 0.05, Fig. 1). We also found significant differences in VEGF levels between groups (20.1 ± 10.0 *versus* 1.6 ± 3.9 pg/ml, respectively in healthy pregnant compared to pre‐eclamptic, *P* < 0.01). Interestingly, six of seven cell cultures (86%) incubated with plasma from pre‐eclamptic pregnant presented VEGFA levels in supernatant below the detection limit. However, only 3 of 10 cell cultures incubated with plasma from healthy pregnant (30%) showed VEGFA levels below the detection limit.

Regarding gene expression, our results were very similar to those reported by Calicchio *et al*. 10. These authors have demonstrated the effect of plasma from pre‐eclamptic pregnant compared to healthy pregnant on gene expression of HUVEC using microarray platform. In their study, 71 genes were up‐regulated and 45 down‐regulated. *VEGFA* was differentially expressed (−1.5‐fold down‐regulated in pre‐eclampsia) between groups. After the microarray analysis, the authors validated those findings using qRT‐PCR, and they found a trend for different *VEGFA* expression between healthy and pre‐eclamptic pregnant (approximately −0.82 reduction in pre‐eclampsia, *P*‐value of 0.08).

Concerning VEGFA (protein) quantification, the limitation in quantification of VEGFA levels in the supernatant (many samples were below the limit of detection) may be related to higher levels of sFLT‐1 found in cultures incubated with plasma from pre‐eclamptic pregnant as sFLT‐1 binds to free VEGFA blocking its effect on endothelial cells *via* membrane‐receptor.

Together these results are very interesting, as miRs play a central role in the regulation of gene expression by binding to 3′UTR of mRNAs, and inducing either mRNA degradation or protein translation repression. Our results demonstrate that both mRNA and protein (VEGFA) are affected (reduced) by plasma from pre‐eclamptic patients in HUVECs. We suggest that part of these effects may be mediated by increased expression of miR‐195‐5p in HUVEC incubated with plasma from pre‐eclamptic pregnant, as demonstrated in human hepatocellular carcinoma 1.

## Conclusion

Endothelial cells incubated with plasma from pre‐eclamptic pregnant show increased miR‐195‐5p expression, which may contribute to decreased expression of VEGFA (gene and protein) and increased anti‐angiogenic status in pre‐eclampsia. Therefore, this miR may be an important target in pre‐eclampsia.

## Funding

This study was funded by Conselho Nacional de Desenvolvimento Cientıfico e Tecnologico (CNPq) and Fundação de Amparo a Pesquisa do Estado de São Paulo (FAPESP‐Brazil).

## Disclosure

The authors reported no conflict of interest.
